# An international medical education collaborative to enhance academic and clinical capacity in South Africa: a six-year review of Discovery-Mass General Fellowship

**DOI:** 10.7189/jogh.16.04041

**Published:** 2026-02-20

**Authors:** Vanessa Bradford Kerry, Brian Allwood, Shrish Budree, Sean Chetty, Neliswa Gogela, Salome Maswime, Sumy Teressa Thomas, Louise C Ivers

**Affiliations:** 1 Center for Global Health, Massachusetts General Hospital, Boston, USA; 2 Division of Pulmonary and Critical Care, Massachusetts General Hospital, Boston, USA; 3 Seed Global Health, Boston, USA; 4 Department of Environmental Medicine, Harvard T.H. Chan School of Public Health, Boston, USA; 5 Department of Medicine, Division of Pulmonology, Stellenbosch University & Tygerberg Hospital, Capetown, South Africa; 6 Department of Paediatrics and Child Health, University of Capetown and Red Cross War Memorial Children's Hospital, Capetown, South Africa; 7 Department of Anaesthesiology & Critical Care, Stellenbosch University, Cape Town, South Africa; 8 Department of Medicine, University of Cape Town & Groote Schuur Hospital, Cape Town, South Africa; 9 Global Surgery Division, Department of Surgery, University of Cape Town, Cape Town, South Africa; 10 Division of Endocrinology, Chris Hani Baragwanath Academic Hospital, Johannesburg, South Africa; 11 Harvard Global Health Institute, Harvard University, Cambridge, USA; 12 Division of Infectious Disease, Massachusetts General Hospital, Boston, USA

## Abstract

**Background:**

The Government of South Africa has prioritised disease prevention and improved healthcare delivery, but the country remains challenged by disease burdens and inequity of resources across the country, including the availability of clinician-specialists to meet public sector and other needs.

**Methods:**

To address the need for more physician specialists in the country, Discovery Health of South Africa collaborated with Mass General Hospital in the USA to create a clinical and research fellowship for South African clinician-scientists. We engaged fellows in semi-structured interviews to chronicle their experience in the programme and its impact and challenges.

**Results:**

Six fellows were awarded the one-year fellowship between 2014 and 2022. Their specialties were in endocrinology, rheumatology, gastroenterology, anaesthesia, and obstetrics and gynecology. All fellows divided their time between research and clinical observership, with programmes individualised for the fellows and mentors in terms of time dedicated to each. The strengths of the programme included formal educational benefits; advanced research techniques, scholarship, and educational opportunities; clinical exposure; strong mentorship; expanded networks; immersive experiences; and accelerated career paths. Weaknesses comprised a relatively short period of study; challenges to reintegration in South Africa; tight budgets to live in the USA; inability to conduct clinical or hands-on practice in the USA; and desire for formal recognition of their time of study in the programme. The fellows all noted the impact of the programme on their own careers- including increased professional opportunities and expanded networks, as well as deepened commitment to and impact in strengthening the capacity of and breadth of service in South Africa’s health system.

**Conclusions:**

Despite challenges to the programme, the fellowship showed the impact of and need for more similar scientific and clinical academic training programmes to build capacity in low- and middle-income countries. With persistent global shortages of health workers, creative solutions that build expertise should be further scaled.

The Government of South Africa has prioritised disease prevention and improved healthcare delivery, specifically targeting initiatives for uniformity, accessibility, and quality within the healthcare system [1]. This has helped advance discovery, research, and clinical care, improving the population’s life expectancy, infant mortality, and other critical markers of improved health, as well as strengthening the health sector’s access to advanced science and care [1,2]. Despite these advancements, the country remains challenged by significant and broad disease burdens, including high HIV and tuberculosis, and inequity of resources reflected in a two-tiered system of public and private care, where most of the population is served by the former [3].

As of 2020, 43 901 medical practitioners were registered in the Health Professions Council of South Africa, with almost half (n = 20 926) being employed in the public health sector. This number is likely to be an overestimation, however, as it includes professionals that have left South Africa, those who have retired, and those who work outside their profession [4]. Furthermore, only 4860 of the 43 901 physicians are designated medical specialists [5], with most primarily working in the private sector [4]. This imbalance creates a considerable challenge to increasing services and access to specialist services for most of the population served by the public sector system. This problem is exacerbated by specialists’ emigration to other, predominantly high-income countries, due to a lack of specialist and clinician-scientist jobs in the public sector in South Africa, as well as better salaries, research opportunities, educational facilities, or working conditions in higher-income countries [6,7]. Taken together, both internal and external brain drain undermines South Africa’s public healthcare system, medical research capacity, and economy.

Global attention has been focused on addressing shortages of all human resources for health to meet the evolving burdens of disease, promote universal health coverage, and improve health security [8]. For South Africa, one of the primary challenges for ensuring progress towards high-quality care has been the training and development of the next generation of clinician leaders, researchers, and professors. In 2009, the Academy of Science of South Africa issued a report calling for increased and long-term investment in academic medicine, research, and clinical-scientific leadership to encourage the country’s excellence in medicine and related research [9]. The report underscored the critical component of training medical practitioners who are ‘fit for purpose’, *i.e.* trained to address the country’s healthcare needs. This would include educating clinician-scientists who will not only be able to work in the ‘trenches’ of the health system, but also to advance research and science in medicine in South Africa.

To help address this need, the Discovery Foundation in South Africa and the Massachusetts General Hospital (Mass General) Center for Global Health joined together to offer one-year academic fellowship awards, the Discovery Mass General Fellowship (DMGF) for clinician-scientists seeking to advance their training and research in academic medicine. The grants aim to ‘to develop the next generation of leaders in academic and clinical medicine in South Africa. The award provides support for mid-career clinical specialists committed to pursuing a career in academic medicine through a fellowship programme that links medical scientists based at South African medical schools, with leaders in clinical science at this world-class medical institution in the United States’ [10]. The programme hosted six fellows between 2014 and 2022 in priority medical specialties who participated in research and clinical observership for one year at Mass General. Here we review the fellows’ experience with the DMGF and its impact on their careers, professional trajectory on return to South Africa, and the identified opportunities and challenges to the programme.

## METHODS

Discovery Health is South Africa’s major medical scheme administrator, which provides administrative and care services for over three million people. Mass General is a 1000-bed academic medical centre based in Boston, Massachusetts, affiliated with Harvard Medical School, and is the most research-intensive hospital in the USA [11]. Aside from research expertise, Mass General receives clinical referrals from across the country, resulting in a mixed patient population of both common and very rare diseases. Through its charitable foundation, The Discovery Foundation, Discovery Health collaborated with Mass General to create the DMGF, which invited South African clinicians to apply for one award per academic year. Applicants were screened by a designated selection committee in South Africa and were required to be recently qualified clinical (medical) specialists who wished to pursue research towards a master’s or doctoral degree. As outlined in the call for proposals, ‘evidence of academic promise and the potential for acquiring new skills, knowledge and expertise that may be transferred to South Africa’ guided the selection. Specifically, preference was given to applicants who worked in full-time research and who presented clearly-defined research projects supported by the host institution, provided they gave evidence of the value of the proposed research and its application to teaching and knowledge generation, and development of, clinical medicine and public healthcare. Eligible applicants were introduced to the DMGF Director at Mass General (VBK) to be connected to potential mentors in their relevant specialty. Mentors and applicants then worked together to create a study path based on which the applicants submitted their candidacy. They were evaluated by the selection committee in discussion with the DMGF Director, at which point a finalist was recommended.

Fellows were awarded a stipend to support travel and time in the USA. There, they worked with a primary mentor who oversaw their research agenda and clinical observerships and who also received a stipend for their time. The fellowship ran for one academic year, generally from July to June, although adjustments were made for applicants during and after the COVID-19 pandemic. Visa laws in the USA preclude foreign medical graduates from participating in direct clinical care, but fellows engaged in in-depth observerships to learn evidence-based clinical practice. Fellows were generally expected to return to South Africa upon completion.

To evaluate their experience of the programme and its impact on their careers, one author (VBK), who served as the director of the programme, and the six fellows (BA, SB, SC, NG, SM, STT) collaborated to review and disseminate an aggregate summary of the fellows’ experiences. All six fellows consented to participate in the research interviews and writing of the article. Based on the nature of the study and structure of the collaboration, institutional review board approval was not sought. One author (VBK) developed an interview guide (**Box 1**) and performed semi-structured interviews with all six fellows between September 2023 and March 2024. Because of the nature of the study and the shared authorship, clarification and elaboration on interview responses were sought iteratively. Responses were recorded by VBK and analysed using an inductive thematic analysis to isolate emerging themes and sub-themes. All reported data was deidentified initially. As co-authors, fellows could revise responses at any time and inclusion of additional and potentially identifying data reported in the manuscript was done voluntarily.

## RESULTS

Six fellows were awarded the DMGF between 2014 and 2022. There was no fellow in 2015, as Mass General was unable to accommodate the eligible applicant proposed by the Discovery screening committee; there was no mentor available in the designated specialty. There was a delay in placing applicants for the programme during the COVID-19 pandemic, and the most recent applicant graduated in the fall of 2022. There were three male and three female fellows. Four were in internal medicine subspecialities, including two in gastroenterology, one in pulmonology/critical care, and one in endocrinology. One was an obstetrician and gynaecologist, and one was an anaesthesiologist with a specialty in critical care and pain medicine (**Table 1**). They came from four academic institutions in South Africa: University of Cape Town, University of the Witwatersrand, University of Kwa-Zulu Natal, and Stellenbosch University.

**Table 1:** Overview of fellows’ specialties and time division while participating in Fellowship

Fellow	Years	Ratio of research and clinical	Returned to South Africa
Hepatology	2014	50:50	Yes
Paediatric gastroenterology	2015–2016	90:10	No
Pulmonary	2017–2018	10:90	Yes
Obstetrics and global health	2018–2019	60:40	Yes
Pain medicine	2019–2020	90:10	Yes
Endocrine	2021–2022	80:20	Yes

All applicants divided their time between research and clinical observership, with programmes individualised for each fellow and mentor in terms of their total percentage of time dedicated to each. Three fellows were focused primarily on research, two split their time evenly between clinical observership and research, and one fellow was almost entirely focused on clinical observership. Applicants spent an equivalent of one academic year (12 months) at Mass General, except for one fellow who spent only nine months when the COVID-19 pandemic forced border closures, and the joint decision was made to ensure they were able to return to South Africa. Five fellows returned to South Africa after the fellowship; a sixth remained in the USA. Of the fellows who returned, all five are employed in the public sector.

Fourteen subthemes related to strengths, weakness, and impacts and opportunities emerged through the qualitative analysis of interviews with the fellows (**Table 2**).

**Table 2:** Themes of strengths, weaknesses, and opportunities for Discovery-Mass General Fellowship

Core theme	Description	Number of fellows reporting
Strengths		
*Advanced research techniques, scholarship and educational opportunities*	Enhanced exposure to advanced research skills, methodology, cohorts, classes, and lectures	5
*Clinical exposure*	Exposed to new clinical techniques, approaches, and quaternary care	3
*Strong mentorship*	Mentorship provided professional growth, opportunity, and networks	5
*Expanded network*	Fellows were exposed to additional collaborators and mentors	4
*Immersive experience*	Dedicated time living and working in the US within a fulltime Fellowship allowed for concentrated and accelerated learning	6
*Accelerated career paths*	Accelerated and increased professional opportunities, research, publications, and advancement	6
Weakness		
*Timeline*	Timeline was not long enough to allow for fully leveraging all the benefits of the programme before returning home	2
*Challenges to reintegration*	Outlined the challenges and barriers to applying their new knowledge and skills on returning or challenges to advancing their careers at home	5
*Budget and funding*	Budget provided was restrictive	6
*Observership*	Limitations to not being allowed to engage in clinical practice but be limited to observing only	5
*Formalised recognition*	Programme and fellows would benefit by issuing a formal certificate or being recognised towards earning a PhD or advanced degree	4
Opportunities		
*Changing mindsets*	Increased sense of confidence, resilience, and career focus	4
*Expanded research collaborations*	Post-fellowship continuation of multinational and/or multi-institutional research networks, strengthened research consortia and opportunities	4
*Advancing medicine and care*	Academic promotion and career, development of new fields of educational tracks, and education of the next generation	6

### Strengths

The strengths of the fellowship included formal educational benefits; advanced research techniques, scholarship, and educational opportunities; clinical exposure; strong mentorship; expanded networks; immersive experience; and accelerated career paths.

#### Advanced research techniques, scholarship, and educational opportunities

The programme created expanded research opportunities for fellows during their time at Mass General through the availability of more advanced, novel techniques to study their specific area of interest and accessibility of advanced and large datasets, allowing them to investigate a broad range of questions that could impact their field. One fellow, for example, learned in-depth coding, data processing, and bioinformatics skills that allowed them to analyse microbiome RNA sequence data. Together with expert mentorship, these skills allowed the fellow to explore several new questions in a genomic microbiome study and to hone them through conversation and thoughtful discourse with colleagues. Through direct work at or collaborations stemming from their time at Mass General, the six fellows published over 99 peer-reviewed articles which have garnered over 8000 citations in aggregate (**Table 3**).

**Table 3:** Publications attributable to Discovery Mass General Fellowship

Fellows	Total	Publications prior to DMGF	Publications during/subsequent	Publications attributable to DMGF	First or last author publications	First or last author attributable to DMGF	Citations*
1	19	1	18	5	3	2	666
2	22	2	20	20	4	2	991
3	159	24	134	64	50	26	4318
4	96	10	86	‡	22	‡	1358
5†	42	23	29	0	24	0	682
6	15	4	11	10	8	5	46
Total	353	64	298	>99	111	>35	8061

DMGF – Discovery-Mass General Fellowship

*Citations determined using ResearchGate.net where all six fellows are listed.

†Fellowship period shortened to nine months by COVID-19 pandemic.

‡Data not available

The time at Mass General also allowed fellows access to additional formal courses and learning opportunities. One fellow participated in several classes at both the Harvard School of Public Health and the Broad Institute, amounting to six formal courses during their stay in Boston. Other fellows were able to access various learning modules and opportunities offered through Mass General and affiliate institutions.

#### Clinical exposure

The clinical observerships of the three more clinically oriented fellows expanded their skill at advanced clinical diagnosis, management, and care. Their exposure to advanced knowledge, evidence-based learning, and clinical innovation and tools allowed them to build specific skill sets with which they could return to South Africa. For one fellow, the time at Mass General enabled them to see the top level of clinical work in their field and to develop sub-specialty expertise not yet available in their country. Through an immersive one-year period, they shadowed a clinician and were repeatedly exposed to procedures, clinical diagnoses, and management in the contexts of both inpatient and outpatient care, creating a powerful clinical learning experience and consolidation of knowledge. Aside from patient care, this fellow was able to observe how the hospital and clinical services were run and care systems organised.

#### Strong mentorship

The DMGF provided a robust mentorship model for the fellows. In detail, the mentors provided deep engagement and expertise, opened doors, and helped fellows expand their skill sets. The fellows valued the relationship with their mentors for the focused support, teaching, and career guidance it provided, and identified it as one of the highlights of the programme.

#### Expanded network

Through their time at Mass General, the DMGF opened networks for the fellows, exposed them to multi-disciplinary academic systems, and fostered engagement with their clinical and research teams. Multiple fellows attended national or international meetings or joined collaborations with broad disciplinary participation. They benefitted from networks within Mass General and Boston, as well as international collaborators to whom they were exposed. However, not all fellows equally leveraged the networking opportunities, as this required them to be proactive and take initiative to maximise the opportunities.

#### Immersive experience

The DMGF afforded important educational, research, and learning space for the fellows who ordinarily manage intense clinical responsibilities in South Africa. As education was the central focus of their experience, fellows had designated time to focus on evidence-based medicine and learning instead of high-volume clinical encounters to meet demand. The fellows also appreciated the breadth of clinical excellence and subspecialist practitioners from whom they could learn at Mass General. Exposure to sub-specialty practice supported bringing new learnings back to South Africa and, in one case, helped introduce a new field of practice in pulmonary.

#### Accelerated career paths

All the fellows’ careers were positively impacted by the Fellowship, which accelerated their learning and career achievements. This impact spanned three categories: increased academic outputs compared to their previous career stage (**Table 3**); greater academic clinical excellence and subspeciality expertise they subsequently utilised in South Africa; or promotions obtained and career advancement on return to South Africa that were facilitated by their time in the USA. Five fellows specifically noted that the dedicated time for research and scholarly pursuit allowed them to produce additional papers and/or build networks that encouraged the advancement of their work and collaborations. The sixth fellow noted their fellowship time was shortened to nine months by the COVID-19 pandemic, which interrupted the plans for their academic deliverables and publications.

### Weaknesses

Interviews with the fellows also identified challenges to the programmes and opportunities to strengthen the experience. These included: relatively short period of study; challenges to reintegration on return to South Africa; very tight budgets to live in the USA; inability to practice clinically or hands-on while in the USA; and desire for more formal recognition of their time of study in the programme.

#### Timeline

The fellows felt that the one-year time allotment was relatively short, as adjustment and integration to the USA and Mass General and processes of on boarding and learning took time. They noted that it takes time to build relationships, build familiarity with the local systems, seek institutional review board approvals, or navigate research study enrollments. Research itself is time consuming, as results may be months away and thus closer to the fellows’ time of departure. One fellow noted that they spent the year trying to fully absorb the research environment and figure out how to best take full advantage of it; by the time they felt comfortable, it was time to leave.

#### Challenges to integration on return to South Africa

Five of the six fellows returned to South Africa; one remained in the USA, but has expressed a desire to return home if the available resources improved to better facilitate track of scientific study. Several of the fellows who returned expressed frustrations with the transition back to South Africa. Those returning to their home institutions felt that there was limited advancement on return nor a facilitated reintroduction. Three noted that they felt that they could not easily implement much of what they learned due to either a lack of resources or lack of support from the administration or academic leadership. Most fellows reported that the academic medical system in South Africa is very hierarchical and paternalistic, which posed challenges for the female fellows bringing innovation and novel expertise. In one case, regulatory barriers challenged a fellow’s ability to access data at their home institution in South Africa, which limited the analysis they were able to conduct while in the USA; they were not able to gain access to the optimal dataset for almost a year. A fellow felt that transferring their work back to South Africa was challenged by a less collaborative approach to work back home; they felt that there was not the same mindset as they experienced in the USA. The one fellow who remained in the USA expressed concern that challenges to funding and the structure of the South African academic system would not provide them with the resources needed to protect the expertise they gained. They would love to return, but do not see a pathway to having a laboratory of their own.

A significant challenge to reintegration shared by several fellows was the discrepancy between their advancement in their year at Mass General and the stagnation and under-resourced context of the South Africa healthcare system. It was difficult to re-acclimate to the hardships and taxed care system in South Africa after exposure to many clinical care options in the USA and the increased human and physical resources. As one fellow stated, ‘After having all the tools and tricks and tips at one’s fingertips and to come back to an overloaded and underfunded system with increased demands becomes very frustrating.’ In South Africa, they noted the simplest things feel disproportionately hard and it can challenge morale: ‘You have changed hugely, but the system has not.’

All the fellows, despite their agreed frustrations at returning, stressed they felt there was opportunity to translate their time in the USA, the skills they learned, and the systems they grew to understand into positive change for their country. They felt that creating more structure for re-entry, as well as the ability to access bridge funding or apply for specific funding to implement new programmes would facilitate them to more directly and fully apply their learning from Mass General into the South African system.

#### Budget and funding challenges

Consistently, fellows felt the funding was tight for the year and led to stress in figuring out how to stretch the available amount. Three fellows had families with children, two were married without children; the former thus had larger challenges financially in terms of covering costs. Three fellows explicitly commented that funding to bridge the return home would have been helpful. These funds could have been used for academic purposes, to support moving their work and studies to their home institutions, and/or to support travel to conferences to follow up on their initial work. These funds could have also served as a bridge while they re-acclimated and determined how to best integrate their learning and time away into new proactive programmes in South Africa.

#### Inability to engage in clinical, hands-on practice

One of the most significant weaknesses of the programme is that fellows could not engage in clinical practice due to licensing regulations in the USA. One fellow did have a brief window of direct clinical care as part of a temporary license agreement which allowed for continuing medical education before it was clarified that USA visa law offer no eligible pathways to allow foreign medical graduates to learn in a clinical setting. Fellows were thus confined to clinical observerships. While the more clinically-focused fellows still noted this as a strong learning opportunity, especially in an immersive and year-long programme, they felt that their overall clinical educational experience would be strengthened by hands-on practice.

#### Formalised recognition

Fellows suggested the Fellowship could be converted to a more formal programme which either contributes to the pathway for receiving a PhD or at least with the award of a recognised certificate which credentials the learning experience. Establishing the Fellowship as a portion of a formal degree or as a discrete certificate pathway would help its impact beyond a one-off year programme with an abrupt end.

### Impact and opportunities

All the fellows agreed that this programme was transformational for their career, emphasising that it should continue, as should any similar programmes, and that the strengths far outweighed the challenges. In addition to the professional growth, collaboration, and expertise built over their year which continued upon return, the fellows highlighted the ways the programme impacted their work and thus medicine in South Africa and beyond.

#### Changing mindsets

Four fellows commented that their time at Mass General and their year learning in the USA’s medical education system increased their confidence and resilience. They felt that they returned to South Africa with a stronger sense of their clinical expertise and understood their potential. All the fellows felt that they developed increased structure and focus for how they wanted to advance their career and the areas for study or clinical practice. One fellow remarked that part of their increased resilience to the challenges of the South African health system came from participating in the broader community created by Discovery Health, including conferences held in South Africa. They noted that the gathering of Discovery awardees to share their work, challenges, and opportunities built a powerful community from which to draw ideas, support, and a vision for South Africa’s potential. The sense of purpose among the fellows was encouraging and gave promise to being able to solve the challenges in South Africa and to think creatively and collaboratively on how to do so.

#### Expanded research collaborations

Several fellows noted that their time at Mass General established powerful multi-national or multi-institution collaborations which have continued to be productive. These collaborations have engaged international consortium of universities (*e.g.* South Africa, USA, UK), and/or the private sector to support innovation. Fellows have been able to continue these collaborations on return including continued publications and research. An example of such is that of one fellow who set up their research protocol to establish a novel South African dataset.

#### Advancing education and care

All fellows noted that their impact on their institutions and South Africa’s medical system was positive on the whole. One fellow, for example, was recruited to start a global programme at a leading South African university; they have been able to leverage their collaborations at Mass General and Harvard University to support the growth of the programme at the institution. Today the fellow is a full professor, leads an academic and research programme, and has developed multiple academic programmes under their purview. They also oversee several learners, including 10 PhDs, 15 master’s students, and 25 others enrolled in an executive leadership course. A second fellow, also a full professor, has gone on to play a leading role globally in establishing and advocating for the new field that recognises the long-term consequences of tuberculosis, including conceptualising and chairing three international symposia on post-tuberculosis lung disease, which has been recognised by the World Health Organization. The fellow initiated a pulmonary hypertension programme, including introducing right heart catheterisation in pulmonology, and has engaged in critical research on the long-term pulmonary hypertension impacts from tuberculosis, building an international reputation and improving diagnosis and management for many patients in South Africa and internationally. A third fellow helped to augment advanced hepatology in South Africa, playing a critical role in education and training of physicians. A fourth fellow is a department chair and involved in national and regional consortia and societies to advance care.

## DISCUSSION

**Figure 1:**
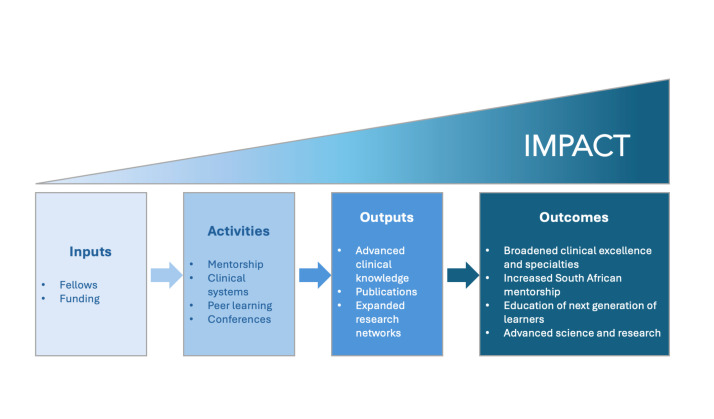
Conceptual framework for the Discovery-Mass General Fellowship.

The DMGF showcases the potential scientific and clinical academic training programmes in building capacity in low- and middle-income countries. The programme was designed to support clinician-scientists to expand their expertise and exposure to novel skills, networks, and systems which could advance their careers, accelerate scientific research, expand clinical options, and advance the health sector in South Africa (**Figure 1**).

With persistent global shortages of health workers and/or imbalances between human resources in public and private sectors, as seen in South Africa, programmes like the DMGF that build expertise should be further scaled. Their results could not only expand the numbers of much-needed trained specialists, but also innovative cross-context collaborations and advancement of scientific research to meet evolving burdens of disease. It is notable that all five of the fellows who returned have remained in the public sector, where they continue to provide care and teach. The benefits of such investment could extend beyond South Africa; the impact of infectious and other diseases, for example COVID, underscore the compelling rationale to develop scientific expertise and research partnerships between high and low/middle income countries. As companies look to run clinical trials, develop local manufacturing or build markets, training specialists that are competent in supporting scientific methods could be helpful to identify and engage local sites. Expertise developed in South Africa for critical scientific and clinical advancement for drug trials, vaccines, or new research will have global impact.

While training costs in the USA are comparably higher [12,13], the highly focused, clinically specialised education opportunities are not yet readily available in South Africa. While the Fellowship demands a large up-front investment, it could achieve long-term savings, as the fellows can help build the specialty capacity in South Africa. These efforts require continued infrastructure and other programmatic support to ensure the specialty’s growth and continuity in the country.

Despite the challenges, the DMGF afforded important educational, research, and learning space for fellows. The shortage of clinician specialists in South Africa puts immense pressure on clinical care providers to move rapidly through clinical encounters, leaving little room for learning or extended thinking about individual patients. System pressures also lead many physicians to be a ‘jack of all trades’, limiting the time, ability, and opportunities to sub-specialise. The time away from intense clinical responsibility enhanced their learning; they could focus on evidence-based medicine and how to integrate it into patient care, and not just move rapidly through clinical care to meet demand.

Five fellows highlighted the strength of the mentorship in the year at Mass General. Several notable research mentorship programmes have historically targeted capacity building, including the USA’s National Institute of Health’s Fogarty Program [14], Wellcome-funded academic exchange programmes [15], or the UK Royal Society’s FLAIR Fellowships [16], but with many of these being primarily research focused. One of the unique characteristics of the DMGF – its targeting of academic clinicians – offers a rare opportunity to learn not only core research skills, but to benefit from clinical mentorship and modelling. Fellows were assigned to clinical and research mentors – often the same individual – offering a bespoke academic research and clinically focused learning intensive.

Despite its strengths, the programme also faced some significant challenges. Issues of budget and duration of the Fellowship are more resolvable than more systemic and rooted ones, such as the desire for formalised credit towards an advanced degree, the inability to engage in clinical practice, or the challenges to reintegration on return to South Africa. The latter two are particularly complex. First, there has been growing literature dedicated to the challenges and frustrations of observership and its limitations in education (compared to clinical practice), including, but not limited to, fewer opportunities for structured feedback, barriers to translating observations into clinical skills, and difficulty being immersed in the clinical experience [17–19].

In Massachusetts, foreign medical graduates are eligible for licenses only if engaged in a formal continuing medical education programme. Mass General developed a course to meet these requirements and pathway; however, the inability to engage in clinical practice is a national restriction embedded in visa laws in the USA. There is currently no eligible visa pathway to allow foreign medical graduates to learn in a clinical setting in any circumstance. Changes to the country’s visa system will need to be executed at a federal Congressional level, and there are initiatives aiming to do so.

Second, while all fellows reported that their careers were accelerated through enhanced research, networks, and recognition of their time in the programme, most of them cited challenges that they felt limited the full impact of the DMGF on return. Many of these challenges are well documented in the literature, including a lack of advanced career opportunities [6,20–22], gender barriers [23,24], and weak investments in the public health sector or education [6,7,25,26]. These barriers, therefore, present a possible target for improving the Fellowship and a potential avenue for further research.

The challenges outlined resonate with those that contribute to both internal and external brain drain. While five of six fellows returned to South Africa, all six of them highlighted challenges of working in South Africa and possible interventions which could leverage the DMGF and support its continued impact in the country. These included structured and committed institutional support on return, tackling the hierarchy and paternalism in academic medicine in South Africa, a more innovative approach to education and collaboration, and additional funding to invest in programme or clinical care expansion. While outside the scope of this study, the experience the fellows outlined helps highlight potential opportunities for intervention to support clinician specialist retention in the public sector or the country. The DMGF was designed, in part, to provide experiences and learning, and to establish the fellows as leaders who could advocate for and create the reforms and investments needed in South Africa’ future.

### Limitations

This study tracks the six fellows who participated in the DMGF programme. They were selected purposefully, as they were engaged in academic medical trajectory in South Africa and came from well established, internationally recognised medical and higher education institutions therein. This limits the generalisability of our findings. Furthermore, this study was designed to elicit the observations, experience, and perspectives of the fellows in the Fellowship and its impact on their careers and field upon return home. It does not, therefore, enable a comparison of how their careers may have advanced without the programme. While this would require a dedicated study, the literature supports the existence of significant challenges faced by similar clinician-scientists and physicians seeking specialisation [12,21,22,25,27]. Finally, the study design does not allow for the characterisation of the barriers to scaling the programme or similar ones; this was outside of the scope of our research and would require a different research approach. Our results may be further limited by the voluntary nature of the questionnaire and a collaborative authorship; fellows may have been more reserved in their critiques or positive in their praise in the interviews by the fellowship director (VBK). Additionally, given the limited number of fellows, it was challenging and at times unrealistic to chronicle the experiences and maintain complete anonymity of those involved. While all fellows opted to participate and report on the programme, its impact and its challenges, and no fellows reported concerns in doing so, the lack of complete anonymity may have unknowingly limited their answers and led to bias.

## CONCLUSIONS

South Africa has prioritised developing its human resources for healthcare by training clinician-scientists and specialists as a pillar of achieving universal health coverage, improving population health and downstream societal gains. As the country enacts its human resources for health strategies, there is an opportunity to leverage academic partnerships, private sector investment, and public sector engagement to harness the scientific and clinical capacity of leading academic clinicians in South Africa in order to strengthen the country’s health system. The DMGF is one such example of an initial investment to build this capacity, but will require continued support from partners in South Africa to ensure its full potential, including ensuring these services to the public sector. Such investments in human resources for health are much needed to tackle the growing burdens of disease from climate change and continued challenges like HIV, preventable maternal and infant deaths, and difficulties with access to basic primary care needs.

Box 1 **Semi-structured interview guide.**

Name

Degrees

Dates at MGH

Background education prior to admission for Discovery-Mass General Fellowship

Medical studies

Research years

Specialty

Academic focus while here?

*How much was clinically oriented? Research? Did you have a preference?*

*What influenced how your programme of study was designed.*

Strengths of programme (open-ended)

Weakness of programme (open-ended)

Areas of growth/opportunity (open-ended)

Did your work and area of study continue after leaving MGH?

*If yes, what facilitated this? If not really, what were barriers?*

Partnerships developed (*e.g.* lasting collaborations, define between whom and how developed)

*What products of this (these) partnerships(s) have emerged (abstracts, papers, research, talks etc.)?*

Papers produced (get citations and PDfs)

Conferences attended (name, specialty, and dates)

*Did you have a speaking or formal role?*

Talks given

Other products

What type of mentorship if any did you have on return?

*Hurt? Help?*

What would have made integration better and/or had more impact?

What would have made the Fellowship experience better?

How do you think programme has had impact if any?

*In your daily practice/career?*

*In your institution?*

*In South Africa?*

*In your field?*

*Elsewhere?*
